# Association between food knowledge and adherence to the Brazilian Food Guide

**DOI:** 10.11606/s1518-8787.2026060006890

**Published:** 2026-05-08

**Authors:** Kamila Tiemann Gabe, Patricia Constante Jaime

**Affiliations:** I Universidade de São Paulo. Faculdade de Saúde Pública. Departamento de Nutrição. São Paulo, SP, Brasil; II Universidade de São Paulo. Núcleo de Pesquisas Epidemiológicas em Nutrição e Saúde. São Paulo, SP, Brasil

**Keywords:** Food Guide, Food and Nutrition Education, Health Literacy, Nutrition Programs and Policies

## Abstract

**OBJECTIVE:**

To investigate the association between food knowledge according to the level of food processing and adherence to the recommendations of the Dietary Guidelines for the Brazilian Population.

**METHODS:**

This study was carried out with a subsample of the NutriNet-Brazil cohort based on quotas (n = 1,053). Adherence to the Guide was assessed using a scale of dietary practices (24 items), which generates a score ranging from 0 to 72. To assess knowledge, participants indicated on a scale of 1 to 10 how healthy they considered each item on a list of 12 foods in four categories (fruit, meat, dairy products and grains), including three groups from the Nova food classification: fresh or minimally processed (G1); processed (G3); and ultra-processed (G4). To generate the score (0 to 8), two points were assigned in each category when option G1 was evaluated as healthier than G3, and both as healthier than G4. In the analysis, sample weighting was used to bring the distribution of the sample closer to the profile of the Brazilian population. The association between the knowledge score and adherence to the Guide was tested using linear regression with adjustment for sociodemographic, socioeconomic and behavioral variables.

**RESULTS:**

The mean score for adherence to the Guide was 43.1 (SD = 9.29) and the mean score for knowledge was 5.4 (SD = 1.2). In the crude model, for every one point increase in knowledge, adherence to the Guide increased by an average of 0.8 points (p = 0.002). The association remained significant in the adjusted model, but the coefficient was reduced to 0.6 (p = 0.020) with the inclusion of socioeconomic variables in the model.

**CONCLUSION:**

Greater knowledge to recognize ultra-processed foods as less healthy is associated with greater adherence to the dietary practices recommended by the Dietary Guidelines for the Brazilian Population, reinforcing the importance of disseminating the recommendations.

## INTRODUCTION

The Dietary Guidelines for the Brazilian Population (hereinafter called Guide), published in 2014, has as one of its principles the promotion of autonomy for healthy food choices. However, it also recognizes that exercising this autonomy depends on overcoming potential barriers to achieving the recommendations, including access to reliable information about healthy eating. According to the Guide, the massive presence in the media of contradictory or pseudo-scientific content about food, often conveyed as a veiled form of advertising for ultra-processed foods, can confuse and hinder healthy food choices^1^.

Since the publication of the Guide, the Brazilian Ministry of Health has invested in disseminating its content, mainly through the production of materials such as booklets, folders and educational videos^2^. Although public policies based on the Guide have been implemented, such as the ban on the sale of ultra-processed foods in institutional spaces of the Ministry of Health in 2015 and the change to the school feeding menus law in 2020, implementation actions in the sphere of disseminating its recommendations have so far been more numerous^2^.

Efforts to disseminate the messages of the Guide are important not only so that its content reaches the population, but also because of the innovative approach presented, which differentiates it from the previous version published in 2006 and from the traditional format of food guides^3^. For the first time, the Guide introduced recommendations based on the Nova food classification, which divides foods into four categories according to the level of processing: fresh and minimally processed, culinary ingredients, processed and ultra-processed^4^. Thus, most of the materials produced so far and made available to the population have sought to address the concept of each of these Nova groups, instruct on how to identify them and suggest strategies on how to adhere to the document’s golden rule: “Always prefer fresh or minimally processed foods and culinary preparations to ultra-processed foods”^1,2^.

On the one hand, evidence shows that public health interventions centered on disseminating information are insufficient to induce a significant improvement in the population’s diet, as they require a high level of individual agency^5–7^. Moreover, this type of initiative, when isolated, tends to be less effective among socioeconomically vulnerable groups, increasing health inequalities^8–11^. One reason for this is that greater knowledge will compete with other barriers to healthy eating, such as exposure to unhealthy food advertising, or physical and financial access to food. On the other hand, it is possible that actions aimed at raising public awareness, even if they affect different segments of the population in a heterogeneous way, will help to stimulate popular demand and support for policies to tackle structural obstacles.

Given the efforts made by the Brazilian government to disseminate the recommendations of the Guide, investigating this issue can provide information on the effect of these actions. This study examined whether greater knowledge of food according to the level of food processing is directly associated with adherence to the recommendations of the Dietary Guidelines for the Brazilian Population, as well as the extent to which this effect is explained by socioeconomic, sociodemographic, and behavioral characteristics.

## METHODS

This is a cross-sectional study carried out with a sub-sample of participants from the NutriNet-Brazil cohort coordinated by the Center for Epidemiological Research in Nutrition and Health at the University of São Paulo (NUPENS-USP), which aims to investigate the relationship between diet and morbidity and mortality from chronic non-communicable diseases in Brazil. The study is carried out entirely through a digital platform and relies on the voluntary participation of people aged 18 and over living in Brazil, who answer questionnaires about health and diet sent out regularly every three to four months.

A minimum sample of 1,224 individuals was drawn to answer a scale of dietary practices used in this study as a measure of adherence to the Guide. This number was distributed into quotas according to gender, education level, and region, considering the proportions observed in the 2010 demographic census of the Instituto Brasileiro de Geografia e Estatística (Brazilian Institute of Geography and Statistics)^12^. The desired number in each quota ranged from three, in the “men from the North region with higher education” quota, to 232, in the “women from the Southeast region without higher education” quota. Thus, 2,145 responded to the scale, filling all the quotas. Of these, those who also answered the Nova-Conhecimento (n = 1,245), a tool used to measure knowledge about food according to the level of processing, applied in another follow-up of the NutriNet cohort, were included in this study. The Guide adherence scale and the Nova-Conhecimento were completed by the individuals included in the study between February 2021 and February 2022. Finally, individuals with missing information on covariates were excluded, totaling a final sample of 1,053 individuals.

Adherence to the Guide was measured using a scale that assesses adherence to the dietary practices recommended in the document^13,14^. It is an instrument with 24 four-point Likert-type items (“never”, “rarely”, “often”, “always”). Four dimensions of the Guide are included in the scale: 1) planning meals, giving preference to healthy foods and sustainable practices; 2) household organization to carry out activities that involve preparing and consuming food, including sharing tasks; 3) adopting appropriate ways of eating, in terms of the environment and regularity with which meals are eaten; and 4) adhering to practices that mark the consumption of ultra-processed foods, such as replacing lunch or dinner food with snacks and consuming snacks between meals, a dimension called food choice. The score on the scale was calculated by the simple sum of the answers provided for each item, with “never” = 0, “rarely” = 1, “often” = 2, and “always” = 3 for the direct items (planning and home organization dimensions), or the opposite for the inverted items, where the answer “never” represents the most appropriate practice and therefore receives a score of 3 (eating habits and food choice dimensions). Thus, the score can vary from 0 to 72^13,14^.

Food knowledge according to the level of processing was measured using Nova-Conhecimento^15^, a version adapted and validated for Brazil of the FoodProK tool^16^, developed by Canadian researchers, based on the NOVA classification of foods. In Nova-Conhecimento, respondents are asked to rate how healthy they consider a sequence of 12 foods divided into four categories (fruit, meat, dairy products, and cereals). Images of each of these foods are presented individually along with a scale of 1 to 10 on which the evaluation is recorded. Packaged foods are accompanied by a nutritional table and list of ingredients. Within each of the four categories, a food belonging to each of the following food groups from the Nova classification is presented: fresh and minimally processed foods (G1), processed foods (G3), and ultra-processed foods (G4)^15^.

To generate the knowledge score, it was checked whether the scores given for each food were in the correct order of healthiness according to the NOVA classification, i.e. G1>G3>G4. Two points were computed in each category when respondents rated the fresh or minimally processed product as healthier than the processed product and both as healthier than the ultra-processed product; one point was computed when only one item was in the correct position. Thus, the total score ranged from 0 to 8 (maximum of 2 points per category).

The selection of items to make up Nova-Conhecimento was based on the frequency and representativeness of items consumed in Brazil according to the National Household Budget Survey (2017–2018). This adaptation involved stages of content validation, pre-testing, discriminant validation, where two groups – undergraduate nutrition students and graduate students - completed the tool and had their scores compared; and convergent validity, assessing the association between the knowledge score and consumption of ultra-processed foods. The tool proved to be valid, given that nutrition students had significantly higher scores than undergraduates and that the score was inversely associated with consumption of ultra-processed foods, as expected^15^.

### Covariates

The following sociodemographic and socioeconomic variables were included in the study: gender (male/female); age (categorized as “18 to 29 years”, “30 to 39 years”, “40 to 59 years” and “60 years or older”); macro-region of the country (North, Northeast, Central-West, Southeast and South); level of education (categorized as “up to complete elementary school”, “complete high school” and “complete higher education or more”); race/color (“white”, “black or brown”, “other or not informed” (the “yellow” and “indigenous” categories were grouped with the “not informed” category due to the low sample size); socioeconomic classification according to the Brazil Criteria of the Brazilian Association of Research Companies^17^ (“A”, “B1”, “B2”, “C1”, “C2”, and “DE”; classes A and B1 and C2 and DE were grouped together in this study); and having private health insurance (yes or no).

Moreover, the number of hours per day of recreational screen use was included in the study, taking into account its association with exposure to food advertising, which could negatively affect the relationship between knowledge and adherence to the Guide^18,19^. The following questions were answered by the participants: “How many hours a day do you usually watch TV?”, “How many hours a day do you usually use a computer/tablet recreationally (to browse social networks such as Facebook/Instagram, to play games, to watch movies, or for similar uses)?” and “How many hours a day do you usually use a cell phone recreationally (to browse social networks such as Facebook/Instagram, to play games, to watch movies, or for similar uses)?”. Time intervals were provided as response options. The total number of hours of exposure to screens was calculated by taking the midpoint of each alternative as the time reported “Less than 1 h” = 30 min; “1 to 2 h” = 1 h 30 min; “2 to 3 h” = 2 h 30 min; “3 to 4 h” = 3 h 30 min; “4 h or more = 4 h 30 min” and “Do not watch TV/use computer/tablet/cell phone recreationally” = 0 h.

### Statistical Analysis

As this was a convenience sample with losses in relation to the initially planned distribution, sample weights were created to bring the profile of the sample closer to that of the Brazilian population in terms of gender, education, and region. This procedure aimed to minimize a potential collision bias in the association analysis, which concerns the observation of spurious associations that occur when both the independent variable of interest and the dependent variable are causes of a third variable not analyzed^20^. In this case, the uninvestigated outcome was participation in the NutriNet Cohort study, since both knowledge of and adherence to dietary practices could lead to selection bias. Sample weighting was considered for all the descriptive and association analyses presented.

The distribution of the sample was presented both in its original form, using absolute and relative frequencies, and weighted, using relative frequencies. The analysis of the association between knowledge according to processing level and the dietary practices score was carried out using linear regression adjusted for the covariates. The covariates were grouped into the following blocks, from the most distal to the most proximal in relation to the outcome: sociodemographic factors (gender, macro-region, age, and race/color), socioeconomic factors (schooling, socioeconomic classification, and having private health insurance), and behavioral factors (time of recreational use of screens).

The normality of the dependent variable was analyzed by inspecting the histogram. For the modeling, a univariate analysis was first carried out between the score and each of the covariates studied. Those with a p-value > 0.20 were eligible as adjustment variables within each block. Next, the association with the outcome of the covariates adjusted by the other variables in the block was analyzed. Those that remained with a p-value > 0.20 were kept for inclusion in the final adjusted model. Finally, the crude association between knowledge and practice score was tested, and then adjusted by the behavioral, socioeconomic and sociodemographic blocks, respectively. The homoscedasticity of the residuals of the final model was checked using graphical analysis. Interactions between the knowledge score and each of the variables at the other levels were tested. All the analyses were carried out using RStudio software version 2023.03.1+446^21^.

### Ethical Aspects

The ethics committee of USP’s School of Public Health analyzed and approved the research project under process number 29139220.9.0000.5421.

## RESULTS

The study sample included 1,053 individuals, of whom approximately 55.0% were women, the majority were from the Southeast region (40.2%) and self-declared white (61.7%). The weighting of the sample led to a better balance between the categories of each variable, especially in relation to race/color, socioeconomic classification and whether they had private health insurance. As for schooling, weighting helped to reduce the proportion of people with complete higher education, but not to increase that of people with only primary education (Table 1).

**Table 1:** Distribution of the sample according to study covariates, weighted and unweighted. Sub-sample of participants in the NutriNet Brazil Cohort, 2022 (n = 1,053).

Variable	n	%	% weighted
Total	1,053	100	100
Gender			
Female	579	54.99	53.29
Male	474	45.01	46.71
Age			
18-29	269	25.55	31.30
30-39	296	28.11	23.81
40-59	363	34.47	34.05
≥ 60	125	11.87	10.84
Region			
North	86	8.17	9.87
Northeast	250	23.74	29.60
Central-West	113	10.73	7.37
Southeast	423	40.17	39.75
South	181	17.19	13.41
Race/color			
White	650	61.73	56.48
Black or brown	370	35.14	40.14
Other/not informed	33	3.13	3.38
Socio-economic classification			
A or B1	277	26.31	19.83
B2	302	28.68	24.57
C1	260	24.69	28.09
C2 or DE	214	20.32	27.51
Schooling			
Up to complete primary education	41	3.89	5.51
Up to secondary school	693	65.81	90.20
Complete higher education	319	30.29	4.29
Private health insurance			
No	470	45.28	54.81
Yes	568	54.72	45.19
Time spent using screens for recreational purposes			
Mean (SD)	6.09 (3.05)		5.82 (3.00)

SD: standard deviation.

^a^Yellow, indigenous or not informed.

In the knowledge assessment, G1 foods received the highest scores in the four categories included in the study (fruit, cereals, meat, and dairy products), followed by G3 foods and, finally, G4 foods (Figure). The food category with the lowest average range between processing levels was dairy products, where UHT milk (G1) received an average score of 6.90, very close to the score for Minas cheese (G3), which was 6.50 (Figure). The knowledge score averaged 5.40 (SD = 1.20), based on the average score in each category (fruit = 1.37; meat = 1.43; corn = 1.36; dairy products = 1.24, on a scale ranging from 0 to 2).

**Figure:**
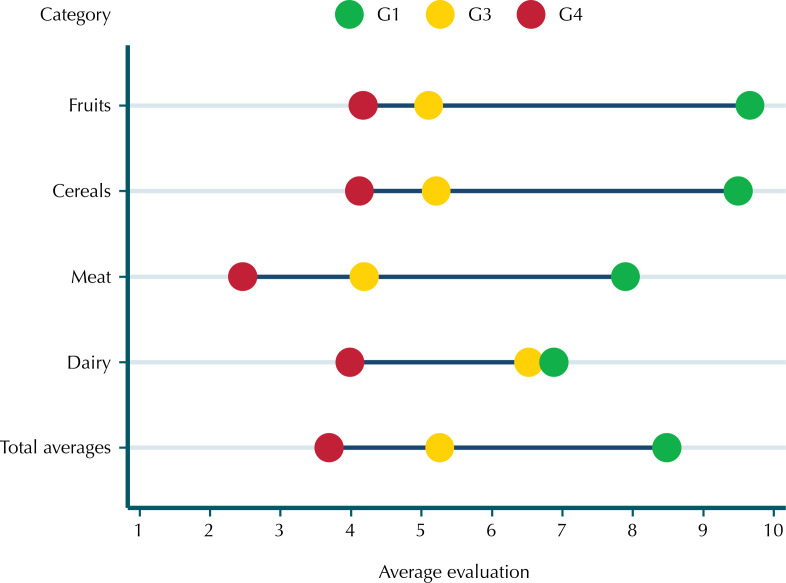
Average rating (1 to 10) given to foods according to category and group in the NOVA classification. Sub-sample of participants in the NutriNet Brazil Cohort, 2022 (n = 1,053).

Fruits: G1: fresh banana; G3: banana jam with added sugar; G4: banana-flavored cereal bar; Cereals: G1: corn on the cob; G3: canned corn kernels; G4: corn snacks; Meats: G1: fresh beef; G3: dried meat; G4: frozen meatballs; Dairy products: G1: UHT whole milk; G3: Minas cheese; G4: strawberry-flavored milk drink.

The average score for dietary practices was 43.10 (SD = 9.29) and ranged from 14 to 70, with a symmetrical distribution. Among the sociodemographic factors, gender (higher among women), race/color (higher among whites compared to blacks and browns), and age (direct association) were associated with the dietary practices score. Among socio-economic factors, the score was lower among people in classes C1 and C2/DE compared to those in classes A/B1, and higher among those with private health insurance. The only behavioral variable, time spent using screens recreationally, was inversely associated with the score (Table 2).

**Table 2:** Average score for adherence to the dietary practices recommended by the Dietary Guidelines for the Brazilian Population according to socioeconomic and sociodemographic variables and time spent using screens. Sub-sample of participants in the NutriNet Brazil Cohort, 2022 (n = 1,053).

Characteristics	Practices score mean or correlation coefficient	Univariate association		Association adjusted by block of variables	
		Beta	p-value	Beta	p-value
Sociodemographic					
Gender					
Male	42.03	-	-	-	-
Female	44.22	2.2	< 0.001	3.0	< 0.001
Age (years)	0.24^a^	0.16	< 0.001	0.17	< 0.001
Race/color					
White	44.31	-	-	-	-
Black or brown	41.38	-2.9	< 0.001	-2.3	< 0.001
Other/not informed^b^	41.98	-2.3	0.14	-0.86	0.6
Region					
North	40.67	-	-	-	-
Northeast	42.92	2.3	0.031	1.0	0.3
Central-West	41.50	0.83	0.5	-0.61	0.6
Southeast	43.57	2.9	0.004	0.42	0.7
South	44.43	3.8	0.002	1.2	0.3
Socio-economic					
Socio-economic classification					
A and B1	46.01	-	-	-	-
B2	44.57	-1.4	0.088	-1.1	0.2
C1	41.99	-4.0	< 0.001	-3.3	< 0.001
C2 and DE	40.64	-5.4	< 0.001	-4.2	< 0.001
Schooling					
Up to complete primary school	43.80	-	-	-	-
Complete secondary school	42.81	-0.99	0.4	-2.3	0.070
Complete higher education	47.16	3.4	0.068	-0.04	> 0.9
Private health insurance					
No	41.78	-	-	-	-
Yes	44.74	3.0	< 0.001	1.7	0.007
Behavioral					
Screen use (hours/day)	-0.27^a^	-0.84	< 0.001	-0.84	< 0.001

^a^Correlation coefficient.

^b^Yellow, indigenous or not informed.

Knowledge was associated with the eating practices score regardless of the adjustment variables. The variable time exposed to screens, when included in the model, showed the same coefficient as in the multiple model, and did not lead to a change in the coefficient of the knowledge score, showing that both variables are independently associated with the score. The inclusion of the socioeconomic block variables adjusted the knowledge coefficient from 0.74 to 0.57, indicating that approximately 20.0% of the effect of knowledge on dietary practices is explained by these factors. The coefficient was not altered by the inclusion of sociodemographic variables, which indicates that the relationship between knowledge and practices is not explained by these variables. The final model explained 16.0% of the variability in the data.

**Table 3:** Crude and adjusted association between food knowledge according to the level of food processing and adherence to the dietary practices recommended by the Dietary Guidelines for the Brazilian Population.

Variable	Crude model		Model 1		Model 2		Model 3	
	Beta	p	Beta	p	Beta	p	Beta	p
Knowledge	0.77	0.002	0.74	0.002	0.57	0.018	0.59	0.017
Screen use (hours)			-0.83	< 0.001	-0.69	< 0.001	-0.66	< 0.001
Socioeconomic class								
A and B1					ref		ref	
B2					-0.50	0.5	-0.17	0.8
C1					-2.6	0.002	-1.90	0.017
C2 and DE					-3.0	< 0.001	-2.00	0.023
Health insurance					1.4	0.020	0.81	0.2
Gender							2.40	< 0.001
Age (years)							0.15	< 0.001
Race/color								
White							Ref	
Black or brown							-1.30	0.026
Other/not informed							1.00	0.6

^a^Yellow, indigenous or not informed.

Note: crude model: knowledge score; R2 = 0.009. Model 1: knowledge + time of recreational screen use; R2 = 0.083. Model 2: model 1 + socioeconomic factors; R2 = 0.1059. Model 3: model 2 + sociodemographic factors; R2 = 0.1639.

## DISCUSSION

The central message of the Dietary Guidelines for the Brazilian Population is that fresh and minimally processed foods and their culinary preparations form the basis of the diet and that ultra-processed foods should be avoided. It also presents messages that emphasize a set of practices related to healthier food consumption, such as cooking regularly, eating mindfully, and not skipping meals^1^. This study showed that greater knowledge to recognize ultra-processed foods as less healthy was associated with greater adherence to such practices. This association was independent of the length of exposure to screens and sociodemographic factors such as gender and age, but was partly explained by socioeconomic factors, especially income.

No other study was found that assessed the association between knowledge according to the level of food processing and dietary practices, limiting the possibility of comparing these findings. However, this result is in line with other studies that investigated the association between food and nutrition knowledge and the quality of food consumption, which also found a direct association^22–25^. According to a systematic review, one of the main limitations of studies of this nature is the lack of alignment between the tools used to assess the two variables – knowledge and diet quality – which is a potentiality of this study, since both tools are anchored in the theoretical framework of the Guide^22^.

It is interesting to note that none of the tools adopted in the study, neither the scale of practices nor the Nova-Conhecimento, use the term ultra-processed. Even so, individuals who were able to interpret product information to identify ultra-processed foods as less healthy had greater adherence to practices that have been shown to be associated with lower consumption of these foods^26^. This reinforces the importance of labeling policies that help consumers identify these foods as less healthy, such as warning labels and regulation of nutrition claims.

The results of this study partly validate the efforts made by public authorities to disseminate the Guide’s recommendations through the preparation of educational materials aimed at both the general population and health professionals^2^. On the other hand, the fact that part of the association is explained by socioeconomic factors shows that dissemination is not enough to promote improvements in the food consumption of the population. In line with this, trends in the Brazilian population’s diet over the last ten years show that consumption of ultra-processed foods has evolved in opposite directions according to characteristics that are markers of socio-economic conditions, which may reflect the actions focused on disseminating the recommendations.

When data from the two most recent available population-based individual dietary surveys (2008–2009 to 2017–2018) are compared, it is observed that the caloric share of the diet derived from ultra-processed foods increased during this period at a slower pace than in the previous period (2002–2003 to 2008–2009), which may be a positive reflection of the Guide^27^. However, when this trend is analyzed in a stratified way for socioeconomic and sociodemographic characteristics, it is observed that consumption has only grown among people with lower levels of education and income, and among self-declared black or brown people^27^. Among the richest and most educated, the indicator even showed a slight decline, which possibly explains the slowdown observed in the aggregate analysis. A similar trend was observed in the prevalence of regular consumption of sugary drinks which, between 2013 and 2019, decreased among people in the highest income quintile and increased among those in the lowest quintile^28^.

These data reinforce the importance of actions aimed at increasing the population’s knowledge combined with those that seek to minimize structural obstacles to achieving a healthy diet. Evidence has shown that the most effective actions to promote healthy eating and with the greatest potential to reduce inequalities are those that affect the price of food, such as taxing unhealthy foods or subsidizing healthy foods^29,30^. In 2024, the new list of the national basic food basket was approved, which expanded and diversified the number of fresh and minimally processed items and excluded ultra-processed foods, which guides the reduction or exemption of taxation for these foods^31^.

Finally, this study also showed that the effect of knowledge on adherence to the Guide occurs independently of the length of time screens have been used for recreational purposes. The literature describes that the use of screens is associated with greater exposure to food advertising^18,19^ and lower diet quality^32,33^, which is consistent with the inverse association observed between this variable and the dietary practices score. It would be plausible to assume that exposure to advertising would compete with knowledge when it comes to making decisions about food choices, thus leading to a reduction in the association between knowledge and adherence to the Guide, which was not observed. This could mean that increasing individuals’ knowledge about healthy eating could have a positive effect regardless of how exposed they are to advertising.

On the other hand, the result also suggests that screens have not been used to convey information about healthy eating, as this would lead to a positive interaction between the two variables. Considering that the Brazilian population is one of the most exposed to screens^34^, large dissemination campaigns on healthy eating through digital media could have a positive impact on the promotion of healthy eating.

The main limitation of this study is that the knowledge assessment instrument was answered approximately 12 months after the eating practices scale. It is possible that part of the association found is therefore explained by reverse causality. In other words, people with greater adherence to healthy practices may have been more exposed to content aligned with the Guide and thus increased their knowledge over this period. However, this bias would be present even if the two instruments were applied at the same time. Intervention studies are needed so that this is controlled in the investigation of this association. In addition, as this is a convenience sample from the Nutrinet-Brasil cohort, it is a more educated sample and possibly one with greater knowledge and a higher score for dietary practices. However, procedures for drawing quotas and then weighing the sample were adopted to minimize this selection bias.

On the other hand, this study is relevant because it is the first to explore the association between knowledge and level of food processing and adherence to the Guide. It is also worth noting that validated instruments were used to assess the constructs of interest in this study, both based on the Guide’s concept of healthy eating. The findings of this study may be useful to support the debate on the implementation of the Guide in Brazil, emphasizing that actions to disseminate and spread the recommendations can have a positive impact on the health of the population.

Greater knowledge to recognize ultra-processed foods as less healthy is associated with greater adherence to the dietary practices recommended by the Guide, reinforcing the importance of disseminating the recommendations. Since part of this association is explained by socioeconomic factors, these actions should be combined with public policies aimed at minimizing other obstacles to healthy eating, such as the cost and availability of food.

## Data Availability

The data used in this study can be made available upon request.

## References

[1] Ministério da Saúde (BR), Secretaria de Atenção Básica, Departamento de Atenção Básica (2014). Guia alimentar para a população brasileira.

[2] Gabe KT, Tramontt CR, Jaime PC (2021). Implementation of food-based dietary guidelines: conceptual framework and analysis of the Brazilian case. Public Health Nutr.

[3] Oliveira MS, Amparo-Santos L (2018). Food-based dietary guidelines: a comparative analysis between the Dietary Guidelines for the Brazilian Population 2006 and 2014. Public Health Nutr.

[4] Monteiro CA, Cannon G, Moubarac JC, Martins AP, Martins CA, Garzillo J (2015). Dietary guidelines to nourish humanity and the planet in the twenty-first century: a blueprint from Brazil. Public Health Nutr.

[5] Adams J, Mytton O, White M, Monsivais P (2016). Why are some population interventions for diet and obesity more equitable and effective than others? The role of individual agency. PLoS Medicine.

[6] Adams J, Hofman K, Moubarac JC, Thow AM (2020). Public health response to ultra-processed food and drinks. BMJ.

[7] Mozaffarian D, Angell SY, Lang T, Rivera JA (2018). Role of government policy in nutrition-barriers to and opportunities for healthier eating. BMJ (Online).

[8] Hyseni L, Bromley H, Kypridemos C, O'Flaherty M, Lloyd-Williams F, Guzman-Castillo M (2017). Systematic review of dietary trans-fat reduction interventions. Bull World Health Organ.

[9] Trieu K, McMahon E, Santos JA, Bauman A, Jolly KA, Bolam B (2017). Review of behaviour change interventions to reduce population salt intake. Int J Behav Nutr Phys Act.

[10] Hyseni L, Elliot-Green A, Lloyd-Williams F, Kypridemos C, O'Flaherty M, McGill R (2017). Systematic review of dietary salt reduction policies: evidence for an effectiveness hierarchy?. PLoS ONE.

[11] Hansen KL, Golubovic S, Eriksen CU, Jørgensen T, Toft U (2022). Effectiveness of food environment policies in improving population diets: a review of systematic reviews. Eur J Clin Nutrit.

[12] Instituto de Geografia e Estatística (2011). Censo demográfico 2010: educação e deslocamento.

[13] Gabe KT, Jaime PC (2019). Development and testing of a scale to evaluate diet according to the recommendations of the Dietary Guidelines for the Brazilian Population. Public Health Nutr.

[14] Gabe KT, Jaime PC (2022). Convergent validity and invariance analysis of a scale to measure adherence to eating practices recommended by the Dietary Guidelines for the Brazilian Population. Rev Bras Epidemiol.

[15] Gabe KT, Basseto G, Jaime PC (2025). Adaptação e validação para o contexto brasileiro de uma ferramenta para avaliação do conhecimento em alimentação baseada na classificação Nova. Epidemiol Serv Saude.

[16] Bhavra J, Kirkpatrick SI, Hall MG, Vanderlee L, Hammond D (2021). Initial development and evaluation of the food processing knowledge (foodprok) score: a functional test of nutrition knowledge based on level of processing. J Acad Nutr Diet.

[17] Associação Brasileira de Empresas de Pesquisa (2016). Critério de Classificação Econômica Brasil.

[18] Fleming-Milici F, Harris JL (2020). Adolescents' engagement with unhealthy food and beverage brands on social media. Appetite.

[19] Demers-Potvin É, White M, Potvin Kent M, Nieto C, White CM, Zheng X (2022). Adolescents' media usage and self-reported exposure to advertising across six countries: implications for less healthy food and beverage marketing. BMJ Open.

[20] Cole SR, Platt RW, Schisterman EF, Chu H, Westreich D, Richardson D (2010). Illustrating bias due to conditioning on a collider. Int J Epidemiol.

[21] R Core Team (2022). R: A language and environment for statistical computing.

[22] Spronk I, Kullen C, Burdon C, O'Connor H (2014). Relationship between nutrition knowledge and dietary intake. Br J Nutr.

[23] Taylor MK, Sullivan DK, Ellerbeck EF, Gajewski BJ, Gibbs HD (2019). Nutrition literacy predicts adherence to healthy/unhealthy diet patterns in adults with a nutrition-related chronic condition. Public Health Nutr.

[24] García-Blanco L, Pascual VO, Berasaluce A, Moreno-Galarraga L, Martínez-González MÁ, Martín-Calvo N (2023). Individual and family predictors of ultra-processed food consumption in Spanish children: the SENDO project. Public Health Nutr.

[25] Carbonneau E, Lamarche B, Provencher V, Desroches S, Robitaille J, Vohl MC (2021). Associations between nutrition knowledge and overall diet quality: the moderating role of sociodemographic characteristics-results from the PREDISE Study. Am J Health Promot.

[26] Gabe KT, Costa CD, Santos FS, Souza TN, Jaime PC (2023). Is the adherence to the food practices recommended by the dietary guidelines for the Brazilian population associated with diet quality?. Appetite.

[27] Louzada ML, Cruz GL, Silva KA, Grassi AG, Andrade GC, Rauber F (2023). Consumo de alimentos ultraprocessados no Brasil: distribuição e evolução temporal 2008-2018. Rev Saude Publica.

[28] Santin F, Gabe KT, Levy RB, Jaime PC (2022). Food consumption markers and associated factors in Brazil: distribution and evolution, Brazilian National Health Survey, 2013 and 2019. Cad Saude Publica.

[29] Thomson K, Hillier-Brown F, Todd A, McNamara C, Huijts T, Bambra C (2018). The effects of public health policies on health inequalities in high-income countries: an umbrella review. BMC Public Health.

[30] Lovhaug AL, Granheim SI, Djojosoeparto SK, Harrington JM, Kamphuis CB, Poelman MP (2022). The potential of food environment policies to reduce socioeconomic inequalities in diets and to improve healthy diets among lower socioeconomic groups: an umbrella review. BMC Public Health.

[31] Brasil (2024). Decreto Nº 11.936, de 5 de março de 2024. Dispõe sobre a composição da cesta básica de alimentos no âmbito da Política Nacional de Segurança Alimentar e Nutricional e da Política Nacional de Abastecimento Alimentar. Diario Oficial Uniao.

[32] Tambalis KD, Panagiotakos DB, Psarra G, Sidossis LS (2020). Screen time and its effect on dietary habits and lifestyle among schoolchildren. Cent Eur J Public Health.

[33] Stiglic N, Viner RM (2019). Effects of screentime on the health and well-being of children and adolescents: a systematic review of reviews. BMJ Open.

[34] DataReportal (2023). Digital 2023: global overview report.

