# Supplementary material for: Association between food knowledge and adherence to the Brazilian Food Guide
Source: Rev Saude Publica. 2026 May 8;60:e16. doi: 10.11606/s1518-8787.2026060006890 (PMC13167058; doi:10.11606/s1518-8787.2026060006890)
Supplement: Translation

# Translation: Associação entre conhecimento em alimentação e a adesão ao Guia Alimentar brasileiro

**Authors:** Kamila Tiemann Gabe, Patricia Constante Jaime

**Affiliations:** I Universidade de São Paulo. Faculdade de Saúde Pública. Departamento de Nutrição. São Paulo, SP, Brasil; II Universidade de São Paulo. Núcleo de Pesquisas Epidemiológicas em Nutrição e Saúde. São Paulo, SP, Brasil

**Keywords:** Guias Alimentares, Educação Alimentar e Nutricional, Letramento em Saúde, Programas e Políticas de Nutrição e Alimentação

## Abstract

**OBJETIVO:**

Investigar a associação entre o conhecimento em alimentação segundo o nível de processamento dos alimentos e a adesão às recomendações do Guia Alimentar para a População Brasileira.

**MÉTODOS:**

Estudo realizado com uma subamostra da coorte NutriNet-Brasil baseada em cotas (n = 1.053). A adesão ao Guia foi avaliada por meio de uma escala de práticas alimentares (24 itens), que gera um escore que varia de 0 a 72. Para avaliar o conhecimento, os participantes indicaram em uma escala de 1 a 10 o quão saudáveis consideram cada item em uma lista de 12 alimentos em quatro categorias (frutas, carnes, laticínios e grãos), incluindo três grupos da classificação de alimentos Nova: *in natura* ou minimamente processados (G1); processados (G3); e, ultraprocessados (G4). Para gerar o escore (0 a 8), dois pontos foram atribuídos em cada categoria quando a opção G1 foi avaliada como mais saudável que a G3, e ambas como mais saudáveis que a G4. Na análise, ponderação amostral foi utilizada para aproximar a distribuição da amostra do perfil da população brasileira. A associação entre o escore de conhecimento e a adesão ao Guia foi testada por meio de regressão linear com ajuste para variáveis sociodemográficas, socioeconômicas e de comportamento.

**RESULTADOS:**

A média do escore de adesão ao Guia foi 43,1 (DP = 9,29) e o de conhecimento foi 5,4 (DP = 1,2). No modelo bruto, para cada um ponto de aumento de conhecimento, a adesão ao Guia aumentou, em média, 0,8 pontos (p = 0,002). A associação permaneceu significativa no modelo ajustado, mas o coeficiente foi reduzido para 0,6 (p = 0,020) com a inclusão no modelo de variáveis socioeconômicas.

**CONCLUSÃO:**

O maior conhecimento para reconhecer os alimentos ultraprocessados como menos saudáveis está associado à maior adesão às práticas alimentares recomendadas pelo Guia Alimentar para a População Brasileira, reforçando a importância da disseminação das recomendações.

## INTRODUÇÃO

O Guia Alimentar para a População Brasileira, publicado em 2014, tem como um dos seus princípios a promoção de autonomia para escolhas alimentares saudáveis. No entanto, reconhece-se também que o exercício dessa autonomia depende da superação de potenciais barreiras para o alcance das recomendações, incluindo o acesso a informações corretas sobre alimentação saudável. De acordo com o Guia, a presença massiva em veículos de mídia de conteúdos contraditórios ou pseudocientíficos sobre alimentação, muitas vezes veiculados como forma velada de publicidade de alimentos ultraprocessados, pode confundir e dificultar escolhas alimentares saudáveis^1^.

Desde a publicação do Guia brasileiro, o Ministério da Saúde tem investido em disseminar o seu conteúdo, sobretudo por meio da produção de materiais, como cartilhas, folders e vídeos educativos^2^. Embora políticas públicas baseadas no Guia tenham sido implementadas, como a proibição da venda de ultraprocessados em espaços institucionais do Ministério da Saúde, em 2015, e a mudança da lei dos cardápios da alimentação escolar, em 2020, as ações de implementação na esfera da disseminação de suas recomendações têm sido até então mais numerosas^2^.

Esforços para disseminar as mensagens do Guia brasileiro são importantes não só para que seu conteúdo chegue na população, mas também pela abordagem inovadora apresentada, o que o diferencia da versão anterior publicada em 2006 e do formato tradicional dos guias alimentares^3^. O Guia introduziu pela primeira vez recomendações baseadas na classificação Nova, que divide alimentos em quatro categorias segundo o nível de processamento: *in natura* e minimamente processados, ingredientes culinários, processados e ultraprocessados^4^. Dessa forma, grande parte dos materiais até então produzidos e disponibilizados à população buscaram abordar o conceito de cada um desses grupos da Nova, instruir sobre como identificá-los e sugerir estratégias de como aderir a regra de ouro do documento: “Prefira sempre alimentos *in natura* ou minimamente processados e preparações culinárias a alimentos ultraprocessados”^1,2^.

Por um lado, evidências apontam que intervenções em saúde pública centradas na esfera da disseminação de informações são insuficientes para induzir melhoria significativa na alimentação da população, por demandarem alto nível de agência individual^5–7^. Além disso, esse tipo de iniciativa, quando isolada, tende a ser menos efetiva entre grupos em situação de vulnerabilidade socioeconômica, aumentando desigualdades em saúde^8–11^. Uma razão para isso é que o maior conhecimento competirá com outras barreiras para uma alimentação saudável, como a exposição à publicidade de alimentos não saudáveis, ou acesso físico e financeiro aos alimentos. Por outro lado, é possível que ações educativas, ainda que incidam de forma heterogênea em diferentes segmentos populacionais, contribuam para estimular a demanda e suporte popular por políticas de enfrentamento a obstáculos estruturais.

Dado o esforço realizado pelo poder público brasileiro em disseminar as recomendações do Guia Alimentar, investigar esse tema pode fornecer subsídios sobre o efeito dessas ações. Este estudo verificou se o maior conhecimento em alimentação segundo o nível de processamento dos alimentos está diretamente associado com a adesão às recomendações do Guia Alimentar para a População Brasileira, bem como o quanto desse efeito é explicado por características socioeconômicas, sociodemográficas e de comportamento.

## MÉTODOS

Trata-se de um estudo transversal realizado com subamostra de participantes da coorte NutriNet-Brasil coordenada pelo Núcleo de Pesquisas Epidemiológicas em Nutrição e Saúde da Universidade de São Paulo (Nupens-USP) que tem como objetivo investigar a relação entre alimentação e morbimortalidade por doenças crônicas não transmissíveis no Brasil. O estudo é realizado inteiramente por meio de uma plataforma digital e conta com a participação voluntária de pessoas com 18 anos ou mais que residem no Brasil, as quais respondem questionários sobre saúde e alimentação enviados regularmente a cada três a quatro meses.

Sorteou-se uma amostra mínima de 1.224 indivíduos para que respondessem uma escala de práticas alimentares utilizada neste estudo como medida de adesão ao Guia. Esse número foi distribuído em cotas segundo sexo, escolaridade e região, conforme as proporções observadas no censo demográfico de 2010 do Instituto Brasileiro de Geografia e Estatística^12^. O número desejado em cada cota variou de três, na cota “homens da região Norte com ensino superior”, a 232, na cota “mulheres da região Sudeste sem ensino superior”. Assim, 2.145 responderam à escala, preenchendo todas as cotas. Destes, foram incluídos no presente estudo aqueles que também responderam à Nova-Conhecimento (n = 1.245), ferramenta utilizada para aferir o conhecimento em alimentação segundo o nível de processamento, aplicada em outro seguimento do estudo de coorte. O preenchimento da escala de adesão ao Guia e da Nova-Conhecimento pelos indivíduos incluídos no estudo ocorreu entre fevereiro de 2021 e fevereiro de 2022. Por fim, foram excluídos indivíduos com informações faltantes em covariáveis, totalizando-se uma amostra final de 1.053 indivíduos.

A adesão ao Guia foi aferida por meio de uma escala que avalia a adesão às práticas alimentares recomendadas no documento^13,14^. Trata-se de um instrumento com 24 itens do tipo Likert de quatro pontos (“nunca”, “raramente”, “muitas vezes”, “sempre”). Quatro dimensões do Guia são compreendidas na escala: 1) realização de planejamento da alimentação, dando preferência a alimentos saudáveis e práticas sustentáveis; 2) organização doméstica para realização de atividades que envolvem o preparo e o consumo de alimentos, incluindo o compartilhamento de tarefas; 3) a adoção de modos de comer adequados, em termos de ambiente e regularidade em que as refeições são realizadas; e 4) a adesão a práticas marcadoras do consumo de alimentos ultraprocessados, como substituir a comida do almoço ou jantar por lanches e consumo de snacks entre refeições, dimensão chamada de escolha dos alimentos. O escore na escala foi calculado pela soma simples das respostas fornecidas a cada item, sendo “nunca” = 0, “raramente” = 1, “muitas vezes” = 2 e “sempre” = 3 para os itens diretos (dimensões planejamento e organização doméstica), ou o oposto para itens invertidos, em que a resposta “nunca” representa a prática mais adequada e, portanto, recebe a pontuação 3 (dimensões modos de comer e escolha dos alimentos). Dessa forma, o escore pode variar de 0 a 72^13,14^.

O conhecimento em alimentação segundo o nível de processamento foi medido por meio da Nova-Conhecimento^15^, uma versão adaptada e validada para o Brasil da ferramenta FoodProK^16^, desenvolvida por pesquisadores canadenses, baseada na classificação Nova de alimentos. Na Nova-Conhecimento, os respondentes são solicitados a avaliar o quão saudável consideram uma sequência de 12 alimentos distribuídos em quatro categorias (frutas, carnes, laticínios e cereais). Imagens de cada um desses alimentos são apresentadas individualmente junto à escala de 1 a 10 em que a avaliação é registrada. Alimentos embalados são acompanhados da tabela nutricional e lista de ingredientes. Dentro de cada uma das quatro categorias, um alimento pertencendo a cada um dos seguintes grupos alimentares da classificação Nova é apresentado: alimentos *in natura* e minimamente processados (G1), alimentos processados (G3) e alimentos ultraprocessados (G4)^15^.

Para geração do escore de conhecimento, observou-se se as notas fornecidas para cada alimento estavam na ordem correta de saudabilidade segundo a classificação Nova, isto é, G1>G3>G4. Dois pontos foram computados em cada categoria quanto os respondentes avaliaram o produto *in natura* ou minimamente processado como mais saudável do que o produto processado e ambos como mais saudáveis do que o produto ultraprocessado; um ponto foi computado quando apenas um item estava na posição correta. Dessa forma, a pontuação total teve variação máxima de 0 a 8 (máximo de 2 pontos por categoria).

A seleção dos itens para compor a Nova-Conhecimento foi baseada na frequência e representatividade de itens consumidos no Brasil de acordo com a Pesquisa Nacional de Orçamentos Familiares (2017–2018). Essa adaptação envolveu etapas de validação de conteúdo, pré-teste, validação discriminante, onde dois grupos – estudantes de graduação em nutrição e de licenciaturas – completaram a ferramenta e tiveram suas pontuações comparadas; e validade convergente, avaliando-se a associação entre o escore de conhecimento e o consumo de alimentos ultraprocessados. A ferramenta mostrou-se válida, dado que os estudantes de nutrição apresentaram pontuações significativamente mais altas do que os de licenciaturas e que o escore esteve inversamente associado ao consumo de ultraprocessados, conforme esperado^15^.

### Covariáveis

Foram incluídas no estudo as seguintes variáveis sociodemográficas e socioeconômicas: sexo (masculino/feminino); idade (categorizada em “18 a 29 anos”, “30 a 39 anos”, “40 a 59 anos” e “60 anos ou mais”); macrorregião do país (Norte, Nordeste, Centro-Oeste, Sudeste e Sul); nível de escolaridade (categorizado em “até fundamental completo”, “médio completo” e “superior completo ou mais”); raça/cor (“branca”, “preta ou parda”, “outra ou não informada” (as categorias “amarela” e “indígena” foram agrupadas à categoria “não informada” devido ao baixo número amostral); classificação socioeconômica segundo Critério Brasil da Associação Brasileira de Empresas de Pesquisas^17^ (“A”, “B1”, “B2”, “C1”, “C2” e “DE”; as classes A e B1 e C2 e DE foram agrupadas neste estudo); e possuir plano privado de saúde (sim ou não).

Além disso, incluiu-se no estudo o número de horas diárias de uso telas para fins recreativos, levando em conta sua associação com a exposição à publicidade de alimentos, a qual poderia interferir negativamente na relação entre o conhecimento e a adesão ao Guia^18,19^. As seguintes perguntas foram respondidas pelos participantes: “Quantas horas por dia você costuma ver TV?”, “Quantas horas por dia você costuma usar computador/*tablet* de modo recreativo (para navegar em redes sociais do tipo Facebook/Instagram, para jogar, para ver filmes ou para usos semelhantes)?” e “Quantas horas por dia você costuma usar celular de modo recreativo (para navegar em redes sociais do tipo Facebook/Instagram, para jogar, para ver filmes ou para usos semelhantes)?”. Intervalos de tempo foram fornecidos como opções de resposta. Calculou-se o número total de horas de exposição a telas assumindo-se como tempo reportado o ponto médio de cada alternativa “Menos de 1 h” = 30 min; “1 a 2 h” = 1 h 30 min; “2 a 3 h” = 2 h 30 min; “3 a 4 h” = 3 h 30 min; “4 h ou mais = 4 h 30 min” e “Não assistir TV/usar computador/*tablet*/celular de modo recreativo” = 0 h.

### Análise Estatística

Por se tratar de uma amostra de conveniência com perdas em relação à distribuição inicialmente planejada, foram criados pesos amostrais para aproximar o perfil da amostra ao perfil da população brasileira em termos de sexo, escolaridade e região. Esse procedimento visou minimizar um potencial viés de colisão na análise de associação, que diz respeito à observação de associações espúrias que ocorrem quando tanto a variável independente de interesse quanto a variável dependente são causas de uma terceira variável não analisada^20^. No caso, esse desfecho não investigado seria a própria participação no estudo, já que tanto o conhecimento quanto a adesão às práticas alimentares poderiam induzir a um viés de seleção. A ponderação amostral foi considerada para todas as análises descritivas e de associação apresentadas.

A distribuição da amostra foi apresentada tanto na sua forma original, por meio de frequências absolutas e relativas, quando ponderada, por meio de frequências relativas. A análise de associação entre o conhecimento segundo nível de processamento e o escore de práticas alimentares foi realizada por meio de regressão linear ajustada para as covariáveis. As covariáveis foram agrupadas nos seguintes em blocos, do mais distal ao mais proximal em relação ao desfecho: fatores sociodemográficos (sexo, macrorregião, idade e raça/cor), fatores socioeconômicos (escolaridade, classificação socioeconômica e possuir plano de saúde privado) e fator comportamental (tempo de uso recreativo de telas).

A normalidade da variável dependente foi analisada por meio de inspeção do histograma. Para a modelagem, primeiramente, foi realizada análise univariada entre o escore e cada uma das covariáveis estudadas. Aquelas que apresentaram p-valor > 0.20 foram elegíveis como variável de ajuste dentro de cada bloco. Em seguida, analisou-se a associação com o desfecho das covariáveis ajustadas pelas demais variáveis do bloco. Aquelas que permaneceram com p > 0.20 foram mantidas para a inserção no modelo final ajustado. Por fim, testou-se a associação bruta entre o conhecimento e o escore de práticas, e em seguida ajustada pelos blocos comportamental, socioeconômico e sociodemográfico, respectivamente. A homoscedasticidade dos resíduos do modelo final foi verificada por meio de análise gráfica. Foram testadas interações entre o escore de conhecimento com cada uma das variáveis dos outros níveis. Todas as análises foram realizadas por meio do *software* RStudio versão 2023.03.1+446^21^.

### Aspectos Éticos

O comitê de ética da Faculdade de Saúde Pública da USP analisou e aprovou o projeto de pesquisa sob processo de número 29139220.9.0000.5421.

## RESULTADOS

A amostra do estudo incluiu 1.053 indivíduos, dos quais aproximadamente 55,0% eram mulheres, a maioria era da região Sudeste (40,2%) e autodeclarada branca (61,7%). A ponderação da amostra levou a um melhor equilíbrio entre categorias de cada variável, sobretudo em relação à raça/cor, classificação socioeconômica e se possui plano de saúde privado. Quanto à escolaridade, a ponderação contribuiu para reduzir a proporção de pessoas com ensino superior completo, mas não para aumentar a de pessoas com apenas ensino fundamental (Tabela 1).

**Tabela 1:** Distribuição da amostra segundo covariáveis do estudo, ponderada e não ponderada. Subamostra de participantes da Coorte NutriNet Brasil, 2022 (n = 1.053).

Variável	n	%	% ponderado
Total	1.053	100	100
Sexo			
Feminino	579	54,99	53,29
Masculino	474	45,01	46,71
Idade			
18–29	269	25,55	31,30
30–39	296	28,11	23,81
40–59	363	34,47	34,05
≥ 60	125	11,87	10,84
Região			
Norte	86	8,17	9,87
Nordeste	250	23,74	29,60
Centro-Oeste	113	10,73	7,37
Sudeste	423	40,17	39,75
Sul	181	17,19	13,41
Raça/cor			
Branca	650	61,73	56,48
Preta ou parda	370	35,14	40,14
Outra/não informada^a^	33	3,13	3,38
Classificação socioeconômica			
A ou B1	277	26,31	19,83
B2	302	28,68	24,57
C1	260	24,69	28,09
C2 ou DE	214	20,32	27,51
Escolaridade			
Até ensino fundamental completo	41	3,89	5,51
Até ensino médio completo	693	65,81	90,20
Ensino superior completo	319	30,29	4,29
Plano de saúde privado			
Não	470	45,28	54,81
Sim	568	54,72	45,19
Tempo de uso de telas para fins recreativos			
Média (DP)	6,09 (3,05)		5,82 (3,00)

DP: desvio padrão.

^a^ Amarela, indígena ou não informada.

Na avaliação do conhecimento, os alimentos G1 receberam pontuações mais elevadas nas quatro categorias incluídas no estudo (frutas, cereais, carne e laticínios), seguidos pelos alimentos G3 e, por fim, pelos alimentos G4 (Figura). A categoria de alimentos com menor amplitude média entre os níveis de processamento foi a dos laticínios, em que o leite UHT (G1) recebeu pontuação média de 6,90, bem próxima à pontuação do queijo minas padrão (G3), que foi de 6,50 (Figura). O escore de conhecimento foi em média 5,40 (DP = 1,20), composto a partir da pontuação média em cada categoria (frutas = 1,37; carne = 1,43; milho = 1,36; laticínio = 1,24, em uma escala de variação de 0 a 2).

**Figura:** Média de avaliação (1 a 10) atribuída aos alimentos segundo categoria e grupo da classificação Nova. Subamostra de participantes da Coorte NutriNet Brasil, 2022 (n = 1.053).

Frutas: G1: banana *in natura*; G3: doce de banana com adição de açúcar; G4: barra de cereal sabor banana; Cereais: G1: espiga de milho; G3: milho em grão enlatado; G4: salgadinho de milho; Carnes: G1: carne de boi fresca; G3: carne seca; G4: almôndegas congeladas; Laticínios: G1: leite integral UHT; G3: queijo minas padrão; G4: bebida láctea sabor morango.

O escore de práticas alimentares foi em média 43,10 (DP = 9,29) e variou de 14 a 70, apresentando distribuição simétrica. Dos fatores sociodemográficos, estiveram associados ao escore de práticas alimentares o sexo (maior entre mulheres), a raça/cor (maior entre brancos em relação aos pretos e pardos) e a idade (associação direta). Dentre os fatores socioeconômicos, o escore foi menor entre pessoas das classes C1 e C2/DE em relação àquelas classificadas nas classes A/B1, e maior entre aquelas que possuem plano de saúde privado. Única variável comportamental, o tempo de uso recreativo de telas foi inversamente associado ao escore (Tabela 2).

**Tabela 2:** Média de escore de adesão às práticas alimentares recomendadas pelo Guia Alimentar para a População Brasileira segundo variáveis socioeconômicas, sociodemográficas e tempo de uso de telas. Subamostra de participantes da Coorte NutriNet Brasil, 2022 (n = 1.053).

Característica	Escore de práticas média ou coeficiente de correlação	Associação univariada		Associação ajustada pelo bloco de variáveis	
		Beta	p-value	Beta	p-value
Sociodemográficas					
Sexo					
Masculino	42,03	-	-	-	-
Feminino	44,22	2,2	< 0,001	3,0	< 0,001
Idade (anos)	0,24^a^	0,16	< 0,001	0,17	< 0,001
Raça/cor					
Branca	44,31	-	-	-	-
Preta ou parda	41,38	-2,9	< 0,001	-2,3	< 0,001
Outra/não informada^b^	41,98	-2,3	0,14	-0,86	0,6
Região					
Norte	40,67	-	-	-	-
Nordeste	42,92	2,3	0,031	1,0	0,3
Centro-Oeste	41,50	0,83	0,5	-0,61	0,6
Sudeste	43,57	2,9	0,004	0,42	0,7
Sul	44,43	3,8	0,002	1,2	0,3
Socioeconômicas					
Classificação socioeconômica					
A e B1	46,01	-	-	-	-
B2	44,57	-1,4	0,088	-1,1	0,2
C1	41,99	-4,0	< 0,001	-3,3	< 0,001
C2 e DE	40,64	-5,4	< 0,001	-4,2	< 0,001
Escolaridade					
Até fundamental completo	43,80	-	-	-	-
Ensino médio completo	42,81	-0,99	0,4	-2,3	0,070
Ensino superior completo	47,16	3,4	0,068	-0,04	> 0,9
Plano de saúde privado					
Não	41,78	-	-	-	-
Sim	44,74	3,0	< 0,001	1,7	0,007
Comportamental					
Uso de telas (horas/dia)	-0,27^a^	-0,84	< 0,001	-0,84	< 0,001

^a^ Coeficiente de correlação.

^b^ Amarela, indígena ou não informada.

Frutas: G1: banana *in natura*; G3: doce de banana com adição de açúcar; G4: barra de cereal sabor banana; Cereais: G1: espiga de milho; G3: milho em grão enlatado; G4: salgadinho de milho; Carnes: G1: carne de boi fresca; G3: carne seca; G4: almôndegas congeladas; Laticínios: G1: leite integral UHT; G3: queijo minas padrão; G4: bebida láctea sabor morango.

O conhecimento foi associado ao escore de práticas alimentares independentemente das variáveis de ajuste. A variável tempo de exposição a telas, quando incluída no modelo, apresentou o mesmo coeficiente que apresentou no modelo múltiplo, e não levou a alteração do coeficiente do escore de conhecimento, mostrando que ambas as variáveis estão associadas ao escore de maneira independente. A inclusão das variáveis do bloco socioeconômico ajustou o coeficiente do conhecimento de 0.74 para 0.57, indicando que aproximadamente 20,0% do efeito do conhecimento sob as práticas alimentares é explicado por estes fatores. O coeficiente não foi alterado pela inclusão das variáveis sociodemográficas, o que indica que a relação entre o conhecimento e as práticas não é explicada por essas variáveis. O modelo final explicou 16,0% da variabilidade dos dados.

**Tabela 3:** Associação bruta e ajustada entre o conhecimento em alimentação segundo o nível de processamento dos alimentos e a adesão às práticas alimentares recomendadas pelo Guia Alimentar para a População Brasileira.

Variável	Modelo bruto		Modelo 1		Modelo 2		Modelo 3	
	Beta	p	Beta	p	Beta	p	Beta	P
Conhecimento	0,77	0,002	0,74	0,002	0,57	0,018	0,59	0,017
Uso de telas (horas)			-0,83	< 0,001	-0,69	< 0,001	-0,66	< 0,001
Class. socioeconômica								
A e B1					ref		ref	
B2					-0,50	0,5	-0,17	0,8
C1					-2,6	0,002	-1,90	0,017
C2 e DE					-3,0	< 0,001	-2,00	0,023
Plano de saúde					1,4	0,020	0,81	0,2
Sexo							2,40	< 0,001
Idade (anos)							0,15	< 0,001
Raça/cor								
Branca							Ref	
Preta ou parda							-1,30	0,026
Outra/não informada^a^							1,00	0,6

^a^ Amarela, indígena ou não informada.

Nota: modelo bruto: escore de conhecimento; R2 = 0,009. Modelo 1: conhecimento + tempo de uso recreativo de telas; R2 = 0,083. Modelo 2: modelo 1 + fatores socioeconômicos; R2 = 0,1059. Modelo 3: modelo 2 + fatores sociodemográficos; R2 = 0,1639.

## DISCUSSÃO

O Guia Alimentar para a População Brasileira preconiza que alimentos *in natura* e minimamente processados e suas preparações culinárias formem a base da alimentação e que os alimentos ultraprocessados sejam evitados. Além disso, apresenta mensagens que enfatizam um conjunto de práticas relacionadas a um consumo alimentar mais saudável, tais como cozinhar com regularidade, comer com atenção e não pular refeições^1^. Este estudo mostrou que o maior conhecimento para reconhecer os alimentos ultraprocessados como menos saudáveis foi associado à maior adesão a tais práticas. Essa associação foi independente do tempo de exposição a telas e de fatores sociodemográficos, como sexo e idade, mas em parte explicada por fatores socioeconômicos, sobretudo pela renda.

Não foi encontrado nenhum outro estudo que tenha avaliado a associação entre o conhecimento segundo o nível de processamento dos alimentos e práticas alimentares, limitando a possibilidade de comparação desses achados. Contudo, esse resultado está em acordo com outros estudos que investigaram a associação entre conhecimento em alimentação e nutrição e a qualidade do consumo alimentar, que também encontraram associação direta^22–25^. Segundo uma revisão sistemática, uma das principais limitações de estudos dessa natureza é a falta de alinhamento das ferramentas utilizadas para avaliar as duas variáveis – conhecimento e qualidade da alimentação – o que é uma potencialidade desse estudo, já que ambas as ferramentas são ancoradas no referencial teórico do Guia^22^.

É interessante notar que em nenhuma das ferramentas adotadas no estudo, nem a escala de práticas, nem a Nova-Conhecimento, é utilizado o termo ultraprocessado. Ainda assim, indivíduos que foram capazes de interpretar informações do produto para identificar os ultraprocessados como menos saudáveis tiveram uma maior adesão a práticas que têm se mostrado associadas ao menor consumo desses alimentos^26^. Isso reforça a importância de políticas de rotulagem que ajudem os consumidores a identificar esses alimentos como menos saudáveis, tais como os selos de advertência e regulação de alegações nutricionais.

Os resultados desse estudo em parte validam o esforço realizado pelo poder público em disseminar as recomendações do Guia por meio da produção de materiais educativos voltados tanto para a população em geral quanto para profissionais da saúde^2^. Por outro lado, o fato de parte da associação ser explicada por fatores socioeconômicos mostra que a disseminação não é suficiente para promover melhorias no consumo alimentar da população como um todo. Em linha, as tendências na alimentação da população brasileira nos últimos dez anos apontam que o consumo de alimentos ultraprocessados evoluiu em direções opostas segundo características que são marcadoras de condições socioeconômicas, o que pode ser reflexo das ações centradas no âmbito da disseminação das recomendações.

Quando analisados dados da população entre os dois últimos inquéritos populacionais de consumo alimentar individual disponíveis (período entre 2008–2009 a 2017–2018), observa-se que o percentual calórico da dieta proveniente de alimentos ultraprocessados cresceu de maneira menos acelerada do que no período anterior (entre 2002–2003 a 2008–2009), o que pode ser um reflexo positivo do Guia^27^. No entanto, quando essa tendência é analisada de forma estratificada para características socioeconômicas e sociodemográficas, observa-se que o consumo tem crescido apenas entre pessoas com menores níveis de escolaridade e renda, e entre pessoas autodeclaradas pretas ou pardas^27^. Entre os mais ricos e escolarizados, o indicador apresentou inclusive um leve declínio, o que possivelmente explique a desaceleração observada na análise agregada. Tendência similar foi observada quanto à prevalência de consumo regular de bebidas açucaradas que, entre 2013 e 2019, decaiu entre pessoas no maior quintil de renda e aumentou entre aqueles no menor quintil^28^.

Esses dados reforçam a importância de ações voltadas a aumentar o conhecimento da população sejam combinadas àquelas que busquem minimizar obstáculos estruturais para o alcance de uma alimentação saudável. Evidências têm mostrado que as políticas mais efetivas para promover alimentação saudável e com maior potencial de reduzir desigualdades são aquelas que incidem sobre o preço dos alimentos, tais como a taxação de alimentos não saudáveis ou de subsídio a alimentos saudáveis^29,30^. Em 2024, foi aprovada a nova lista da cesta básica nacional que ampliou e diversificou o número de itens *in natura* e minimamente processados e excluiu os alimentos ultraprocessados, a qual orienta a redução ou isenção de tributação para esses alimentos^31^.

Por fim, este estudo também mostrou que o efeito do conhecimento sobre a adesão ao Guia ocorre de maneira independente do tempo de uso de telas para fins recreativos. É descrito na literatura que o uso de telas está associado à maior exposição à publicidade de alimentos^18,19^ e menor qualidade da alimentação^32,33^, o que é coerente com a associação inversa observada entre essa variável e o escore de práticas alimentares. Seria plausível assumir que a exposição à publicidade competiria com o conhecimento na tomada decisão para a escolha dos alimentos, levando, portanto, a uma redução da associação do conhecimento com a adesão ao Guia, o que não foi observado. Isso pode significar que aumentar o conhecimento dos indivíduos sobre alimentação saudável poderá ter um efeito positivo independentemente do quão expostos à publicidade os indivíduos estejam.

Por outro lado, o resultado também sugere que as telas não têm sido utilizadas para veicular informação sobre alimentação saudável, pois isso levaria a uma interação positiva entre as duas variáveis. Considerando que a população brasileira é uma das mais exposta a telas^34^, grandes campanhas de disseminação sobre alimentação saudável por meio de mídias digitais poderiam ter um impacto positivo na promoção da alimentação saudável.

A principal limitação deste estudo é que o instrumento de avaliação de conhecimento foi respondido aproximadamente 12 meses após a escala de práticas alimentares. É possível que parte da associação encontrada seja, portanto, explicada por uma causalidade reversa. Ou seja, pessoas com maior adesão a práticas saudáveis possivelmente se exponham mais a conteúdos alinhados ao Guia e, dessa forma, teriam aumentado o conhecimento ao longo desse período. Contudo, esse viés estaria presente mesmo que os dois instrumentos fossem aplicados ao mesmo tempo. Estudos de intervenção são necessários para que isso seja controlado na investigação dessa associação. Além disso, por se tratar de uma amostra de conveniência oriunda da coorte Nutrine-Brasil, trata-se de uma amostra mais escolarizada e, possivelmente, com maior conhecimento e maior escore de práticas alimentares. No entanto, procedimentos de sorteio de cotas e, posteriormente, ponderação da amostra, foram adotados para minimizar esse viés de seleção.

Por outro lado, esse estudo é relevante por ser o primeiro a explorar a associação do conhecimento segundo o nível de processamento dos alimentos na adesão ao Guia. Além disso, vale destacar que foram utilizados instrumentos validados para avaliar os constructos de interesse desse estudo, ambos elaborados a partir do conceito de alimentação saudável do Guia. Os achados deste estudo podem ser úteis para embasar o debate sobre a implementação do Guia no Brasil, ressaltando que as ações de disseminação e capilaridade das recomendações podem ter um impacto positivo na saúde da população.

O maior conhecimento para reconhecer os alimentos ultraprocessados como menos saudáveis está associado à maior adesão às práticas alimentares recomendadas pelo Guia, reforçando a importância da disseminação das recomendações. Visto que parte dessa associação é explicada por fatores socioeconômicos, essas ações devem ser combinadas a políticas públicas que visem minimizar outros obstáculos para uma alimentação saudável, tais como o custo e a disponibilidade dos alimentos.

## Data Availability

Os dados utilizados neste estudo podem ser disponibilizados mediante solicitação.
